# Prepupal Building Behavior in *Drosophila melanogaster* and Its Evolution under Resource and Time Constraints

**DOI:** 10.1371/journal.pone.0117280

**Published:** 2015-02-11

**Authors:** Sunitha Narasimha, Sylvain Kolly, Marla B. Sokolowski, Tadeusz J. Kawecki, Roshan K. Vijendravarma

**Affiliations:** 1 Department of Ecology and Evolution, University of Lausanne, Lausanne, 1015, Switzerland; 2 Department of Ecology and Evolutionary Biology, University of Toronto, Toronto, Canada, M5S 3B2; University of Houston, UNITED STATES

## Abstract

Structures built by animals are a widespread and ecologically important ‘extended phenotype’. While its taxonomic diversity has been well described, factors affecting short-term evolution of building behavior within a species have received little experimental attention. Here we describe how, given the opportunity, wandering *Drosophila melanogaster* larvae often build long tunnels in agar substrates and embed their pupae within them. These embedded larvae are characterized by a longer egg-to-pupariation developmental time than larvae that pupate on the surface. Assuming that such building behaviors are likely to be energetically costly and/or time consuming, we hypothesized that they should evolve to be less pronounced under resource or time limitation. In accord with this prediction, larvae from populations evolved for 160 generations under a regime that combines larval malnutrition with limited developmental time dug shorter tunnels than larvae from control unselected populations. However, the proportion of larvae that embedded before pupation did not differ between the malnutrition-adapted and control populations, suggesting that tunnel length and likelihood of embedding before pupation are controlled by different genetic loci. The behaviors exhibited by wandering larvae of *Drosophila melanogaster* prior to pupation offer a model system to study evolution of animal building behaviors because the tunneling and embedding phenotypes are simple, facultative and highly variable.

## Introduction

In many holometabolous insects the feeding larval phase is followed by a non-feeding ‘wandering’ phase prior to pupation, where the larvae cease feeding and leave the feeding site in search of a suitable pupation site [1]. The wandering period is associated with a number of prepupation behaviors and in some species involves building structures within which the insects pupate [2–4]. These pupation structures range in complexity from simple burrows to spectacular hammocks, and they can increase fitness by conferring some protection against adverse biotic and abiotic environmental factors, such as temperature, humidity and natural enemies[2]. However, building behaviors are time consuming and energetically demanding [2]. Thus, when resources are limited, natural selection should favor optimal allocation of time and energy to such building behavior given the trade-offs [5,6]. Such trade-offs might be difficult to study in species with complex constructions, which in the course of evolution have become fully dependent on such structures for survival. Evolution of building behavior would thus be more amenable to experimental study in species where this behavior is simple, facultative and highly variable. Here we describe such a behavior in *Drosophila melanogaster* and show using an evolution experiment [7] that its extent becomes reduced in populations evolutionarily adapted to chronic larval malnutrition, suggesting an evolutionary trade-off between adaptation to nutritional stress and propensity to build.

In their natural habitats, pupation sites of *D*. *melanogaster* larvae vary widely; some larvae pupate on the fruit, while others, prefer pupating in the soil [8]. Furthermore, factors such as presence of conspecifics, moisture, texture and chemical properties of the substrate influence larval pupation site choice [9,10]. In the laboratory, *Drosophila* larvae typically wander and pupate on the walls of the container because the bottom of the rearing vial or bottle is usually entirely covered by food [11]. The distance that larvae pupate from the surface of the food ‘pupation height’ is a polygenic trait that responds effectively to bidirectional selection [12,13] and is influenced by light, temperature, humidity, pH, density and parasitism [14–17]. When laboratory culture conditions are enriched with horizontal semi-natural arenas (soil, agarose, etc) around the feeding medium, larvae prefer to wander and pupate in these arenas [13,18]. Interestingly, in agarose arenas larvae either pupate on the surface of the agar or burrow through the agar and pupate embedded in the agar with their posterior end embedded in the agar [19]. The incidence of embedding is significantly reduced in constant darkness and has responded within six generations to bidirectional selection [20]. However, the behavioral sequence leading to embedding behavior has not been reported [19,20].

In the present study, we describe the behaviors leading to ‘embedding’ in *Drosophila melanogaster* larvae. It involves two distinct phases: first, the digging of a tunnel and second, the preparation of the tunnel exit for embedding of the pupa. Our experimental assay allows wandering larvae to exhibit tunneling and embedding behavior in agar, which is quantifiable by the ‘extended phenotype’, the tunnels that the larvae leave behind. Given the high costs of larval movement [21] and the physical resistance of the substrate, this building behavior is likely both energetically costly and time consuming [22]. Because larvae do not feed during the wandering period, prepupation behaviors such as tunneling depend on energy stores acquired during the foraging period of larval development (i.e., during the first, second and half of the third instar). The wandering period of larval development is relatively short and is completed within a matter of hours. Resource scarcity during larval development and time constraints may therefore lead to trade-offs between this building behavior (the tendency to tunnel and embed) and other life history traits, favoring a reduction in building activity [5,6,22].

We test this prediction using experimental evolution [7]. We use six *D*. *melanogaster* populations which in the course of 160 generations were forced to develop on extremely poor-quality larval food (see Methods) [23]. Their adaptation to this larval malnutrition regime has been manifested in improved survival and faster larval growth on the poor food [23]. Even when raised on standard food, these malnutrition-tolerant populations show faster development [23], lower critical-size for pupation [24], increased larval competitive ability [25], ‘sitter’ like foraging behavior [26] and increased propensity for larval cannibalism [27], and they are smaller and less fecund as adults [23]. Here we test if the nutritional and developmental time constraints imposed on these populations led to the evolution of reduced tendency to tunnel and embed, comparing them to control populations, which had evolved under standard food conditions.

## Results

### Building behavior

**Fig 1:**
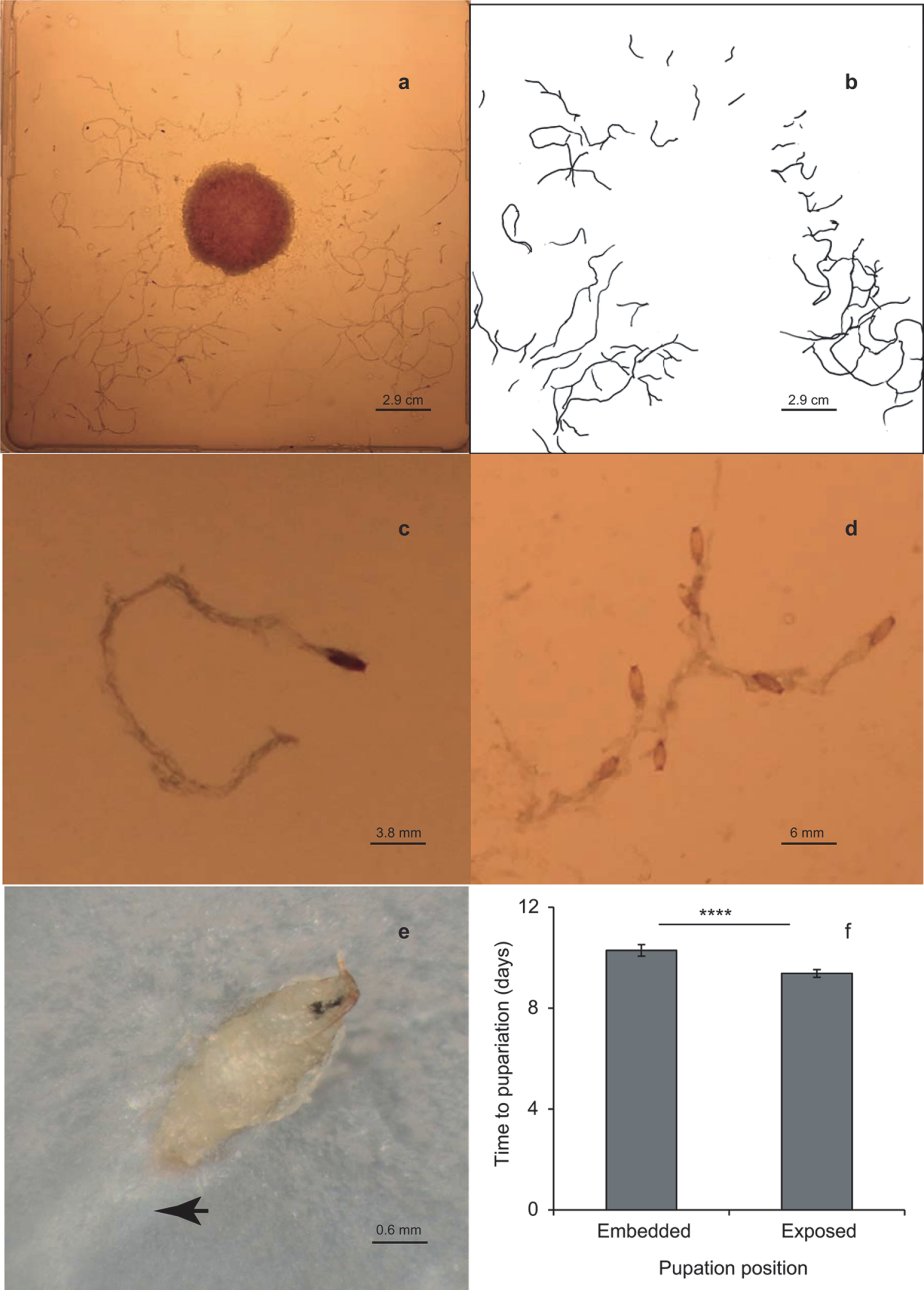
Larval building behavior. (a) Assay plates with a central food zone surrounded by an agarose arena where larvae pupated; tunnels built by embedding larvae are revealed by backlighting the plate. (b) An example of larval tunnels traced onto a transparency and digitized for quantification. (c) A simple tunnel with solitary pupa. (d) A complex tunnel formed by several individuals adding branches to existing tunnels. (e) A pupa embedded in the agar surface, the arrowhead points to the tunnel. (f) Developmental time from egg laying to pupariation for embedded and exposed pupae (the Valais strain; means ± 95% CI, ‘****’ indicates *p* < 0.0001, *N* = 8 plates with 59–83 pupae per plate).

In our custom-made culture plate set-up (Fig. 1A, see Methods), larvae close to completion of their development exhibit wandering behavior. They leave the centrally located food patch and range widely over the surface of the agar plate with the vast majority of larvae pupating well away from food. Many of these larvae dug elaborate tunnels through the (non-nutritional) agar using their mouth hooks (Fig. 1A,C,D). These tunnels can be easily traced, digitized and quantified (See Methods; example Fig. 1B). This tunneling behavior concluded with the larva preparing the tunnel exit for subsequent embedding of the pupa (Fig. 1E). As a tunneling larva reached the surface, it retained its posterior end within the tunnel and repeatedly rasped its mouth hooks all around the exit churning the solid agar into a mushy paste for over an hour, before finally pupating in it (Fig. 1E; S1 Video). Since these tunnels are dug in non-nutritious agar by wandering larvae that have ceased feeding, they are unrelated to the tunnels incidentally created in food while foraging. Most larvae dug their tunnels independently and pupated solitarily (Fig. 1C). However, it was common to find larvae entering and extending pre-existing tunnels dug by other larvae, and pupating communally forming a branched labyrinth of tunnels (Fig. 1D). This observation supports an essential characteristic of building behavior that, in addition to building new structures, individuals must also be able to extend already existing structures [2]. These behaviors were unambiguously observed in several *D*. *melanogaster* strains of diverse origin: CantonS, Valais, as well as in our experimentally evolved six selected and six control populations (see below). The proportion of larvae that pupated by embedding and the average lengths of the tunnels that were dug varied between strains. However, all embedding larvae were observed preparing their tunnel exit in the same way as described above (S1 Video). The building behavior in one of the control population was most pronounced on agar substrates of standard concentration (2% w/v) and decreased at concentrations above or below this (S1A, Fig. B), We confirmed this pattern with one of the selected populations; again the frequency of embedding and tunnel length were the highest on 2% agar (S1C, Fig. D) and we thus used 2% agar plates for all the subsequent experiments.

We quantified larval developmental time as the time from egg laying to pupariation (i.e., eversion of spiracles and formation of the pupal case which precedes pupation [28]). Larvae that pupated by embedding in the agar pupariated on average one day later than larvae that pupated on the agar surface (Fig. 1F; *F*_1, 7_ = 115.4, *p* < 0.0001). This implies that tunneling and embedding activity delays metamorphosis, or that only larvae whose development has been slower for another reason engage in tunneling. Both interpretations are consistent with the notion that these behaviors are costly in terms of time.

### Experimental evolution of pre-pupation behavior

**Fig 2:**
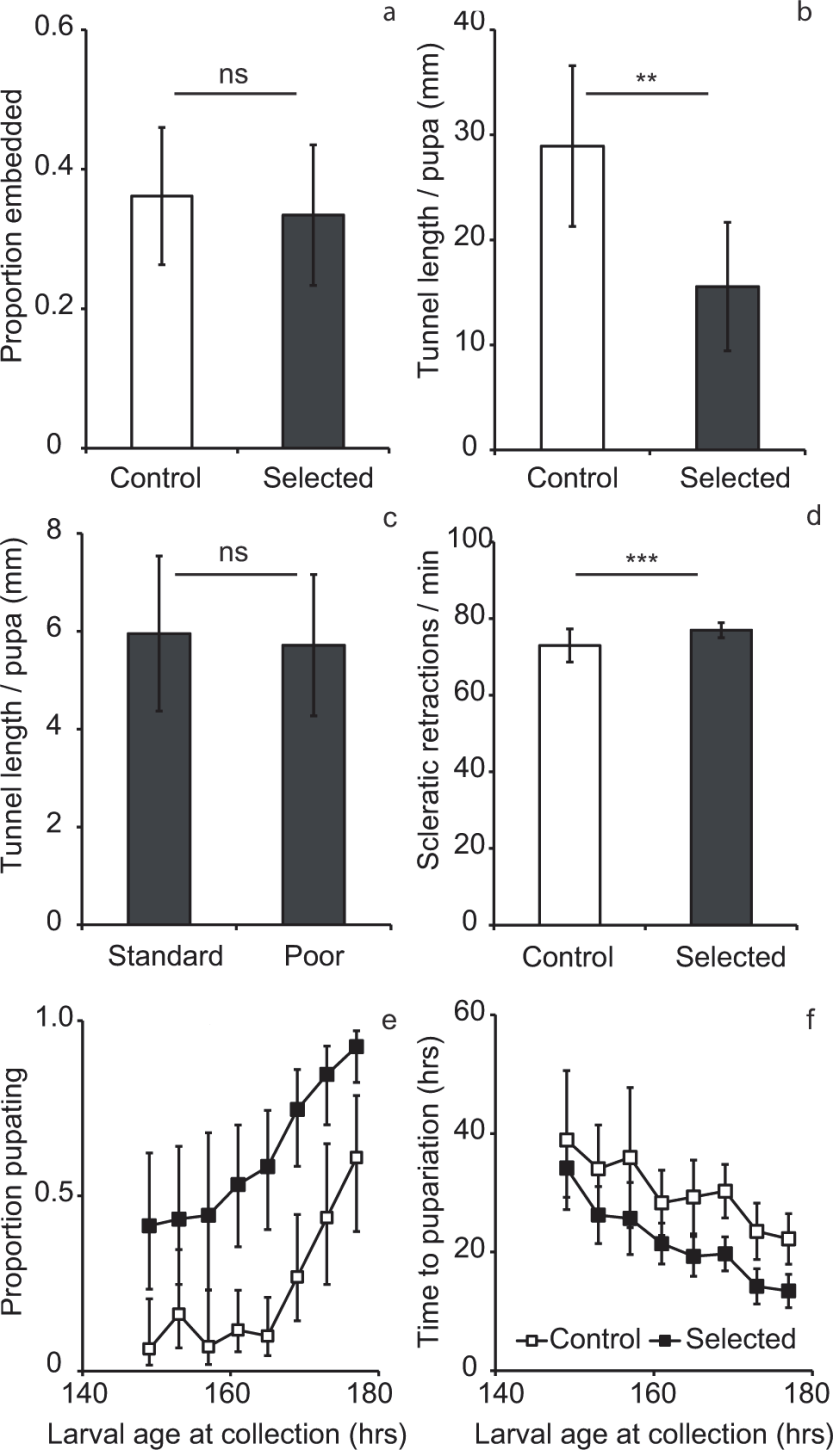
Building behavior traits in malnutrition tolerant selected populations (dark symbols) compared to unselected controls (white symbols). (a) The proportion of pupae that embedded in the agar substrate and (b) the average tunnel length per embedded pupa for the selected and control populations; *N* = 3 assay plates per population. (c) The effect of food quality on average tunnel length per embedded pupa for the selected populations. (d) Tunneling effort measured as the rate of scleratic retractions per minute, *N* = 8 larvae per population. (e-f) Assay of the interval between larval wandering forays and pupariation: (e) The proportion of larvae collected outside of food which succeeded to pupariate in the absence of food as a function of age at collection (logistic ANCOVA: slope *F*_1,81_ = 110.8, *P* < 0.0001, regime *F*_1,10_ = 22.9, *P* < 0.001). (f) The time between collection and pupariation as a function of age at collection (ANCOVA: slope *F*_1,67_ = 112.0, *P* < 0.0001, regime *F*_1,10_ = 27.7, *P* < 0.001). In all panels the error bars indicate 95% confidence intervals, in panels a-d ‘ns’ indicates not significant while ‘**’ and ‘***’ indicate *p* < 0.01 and *p* > 0.001 respectively. Except as indicated in panel (c), all larvae used in these assays were raised on standard food.

To test whether evolution under nutrition and time constraints leads to a reduction in tunneling and embedding behavior, we compared these behaviors between the six malnutrition-tolerant selected populations and the six control populations briefly described in Introduction (see Methods for details). Around 70–82% of individuals pupariated in all plates; this proportion did not differ between the evolutionary regimes (selected: 0.74 ± 0.06, control: 0.77 ± 0.04; *F*_1, 10_ = 0.9, *p* = 0.36). Pupation occurred mostly outside the food in both regimes and only occasionally on the food (< 3%) in some plates. The mean distance of the pupa from food did not differ between the selection regimes (selected: 61.0 ± 0.4 mm, control 60.7 ± 0.5 mm; *F*_1, 10_ = 0.002, *p* = 0.97), although it varied among the replicate populations (*F*_10, 22_ = 3.0, *p* = 0.02). Similarly, the regimes did not differ in the proportion of larvae that pupariated embedded in a tunnel versus on the agar surface (Fig. 2A; *F*_1, 10_ = 0.3, *p* = 0.61), even though replicate populations within each regime varied to some degree (*F*_10, 22_ = 4.5, *p* = 0.002). Although the survival of embedded and exposed pupae could not be assayed separately in our set up, 96–99% of pupae eclosed as adults in all plates, with no difference between the regimes (*F*_1, 10_ = 0.07, *p* = 0.8).

Even though the selected and control populations did not differ with respect to the above traits, they showed a striking difference in the tunneling behavior. The combined length of tunnels dug by the selected population was 50% shorter than those dug by control populations, whether one compares the total tunnel length per assay plate (*F*_1, 10_ = 40.7, *p* < 0.0001) or average tunnel length per embedded pupa (Fig. 2B; *F*_1, 10_ = 12.3, *p* < 0.006).

In the above assays, both selected and control larvae were raised on the standard food. Even though the larvae of the selected populations perform well on standard food (see Introduction), one might imagine that their shorter tunnel length was a reaction to the standard food, to which they were no longer fully adapted. To address this possibility we compared the tunnel length of selected populations reared on standard versus poor food. Around 42–47% of the larvae pupated by embedding irrespective of the food quality they were raised on (*F*_1, 10_ = 3.4, *p* = 0.1), and no effect of food rearing quality was found on the tunnel length per embedded pupa (Fig. 2C; *F*_1, 10_ = 0.08, *p* = 0.78). This confirmed that the observed difference in tunnel length between selected and control populations was not a consequence of the selected larvae being raised on a different food substrate than that they encountered in the last 160 generations.

Finding shorter tunnels in selected populations which evolved under chronic malnutrition and a constraint on maximum developmental time might result from the selected larvae putting less effort into digging tunnels, e.g., because they were physically weaker. We quantified tunneling effort by measuring the rate of larval mouth hook retractions while tunneling. The selected larvae showed a 5% higher rate of mouth hook retractions than the control larvae (Fig. 2D; *F*_1, 10_ = 20.0, *p* < 0.001). This suggests that larvae from the selected populations do not exert less effort while digging tunnels than those from the control populations.

Do selected larvae spend less time wandering, and thus have less time for tunneling? To address this question we assayed how soon after initiation of wandering the larvae pupariate. We periodically collected third instar larvae of known age that were found on the assay plates more than 15 mm away from food, transferred them to a petri dish with agar (no food), and recorded the time elapsed until they pupariated. Many of these larvae failed to pupariate, indicating that they had just left food temporarily and were not yet truly in the physiologically-defined wandering phase [28]. As expected, the proportion of the collected larvae that did pupariate (pupariation success) increased with larval age at collection (Fig. 2E), while the average time from collection to pupariation declined (Fig. 2F), reflecting a greater developmental advance of older larvae. For any age at collection, the pupariation success was higher and the time to pupariation was shorter for the selected than control populations (Fig. 2E,F); this again is expected because the selected larvae develop faster [23]. The difference between selected and control lines in time from collection to pupariation (Fig. 2F), is consistent with the former being 14.8 hours more advanced in their development. This is similar to the 11–15 hours difference in developmental time between the selected and control larvae observed in past assays ([23]; R. K. Vijendravarma and S. Narasimha, unpublished results). In a further attempt to separate the putative differences in the length of wandering phase from the effect of attributable to selected larvae being more advanced in their development, we used the logit-transformed mean pupariation success as a proxy measure of developmental stage. When this measure was used as covariate, the difference in the time to pupariation between selected and control populations disappeared (ANCOVA; regime *F*_1,10_ = 0.02, *P* = 0.90; 95% confidence interval on the difference between selected and control populations: [-4.3 h,4.8 h]). Thus, when corrected for the difference in rate of development, larvae from the selected populations do not seem to have markedly more time left to pupariation when they begin wandering than the control populations.

## Discussion

Pre-pupation tunneling behavior first described in this paper is a genetically variable trait, the expression of which—as quantified by the length of tunnels—can quickly evolve in response to larval nutritional conditions. The evolution of shorter tunnel length in our selected populations supports the hypothesis that this behavior is costly in terms of time and/or energy and thus becomes counter-selected if the larvae are forced to develop within a limited time on a nutrient-poor food.

It is difficult to disentangle to what extent the decline in tunneling in the selected population has been driven by its time versus energy costs. The selected populations evolved to develop faster, likely in part due to the fact that the selection regime imposed a 14 day limit on the egg-to-adult developmental time [23]. However, this is mediated at least in large part by their smaller critical size, and thus a shorter foraging period [24]. Our results here suggest that the time between first wandering forays and pupariation (i.e., the time window available for tunneling) is not markedly shorter in the selected than control populations. On the other hand, we have shown that, within a population, the individuals that pupate embedded—and thus make at least short tunnels—have a markedly longer egg-to-pupariation development time, although we do not know if developmental time is correlated with tunnel length. Some environmental factors are known to delay pupariation in *Drosophila* [29] and some other Diptera [1], and one may speculate that tunneling and/or embedding activity acts as a stimulus delaying pupariation. However, the shorter developmental time of embedded pupae may be mediated by slower development during the foraging stage rather than by prolongation of the wandering stage. In either case, if the within-population association between developmental time and embedding had been a major mechanism responsible for the differences in the tunneling behavior between the selected and control populations, selected populations should have evolved a reduced frequency of embedding as a correlated response, which was not the case. Thus, the pressure to save energy remains a plausible explanation for reduction in tunnel length. While the energetic costs of tunneling would be difficult to measure, digging through the relatively sticky agarose is unlikely to be effortless, given that even locomotion on a surface requires a measurable energy expenditure in Dipteran larvae [30] This is supported by the decrease in embedding and tunneling activities on harder agar. A reduction in an energetically costly activity not involved in nutrient acquisition would be consistent with other evolutionary changes in the selected populations that can be interpreted as contributing to frugal use of resources, such as less movement while foraging [26] and a smaller critical size for metamorphosis initiation [24].

While the above arguments invoke direct selection against long tunnels under our selection regime, we cannot exclude that shorter tunnels in selected populations evolved as a correlated response to selection on some other traits, mediated by pleiotropy of the underlying polymorphisms. In particular, we have shown before that the selected populations evolved to move less while foraging [26], reminiscent of the "sitter" phenotype [31,32]. The "sitter"–"rover" phenotypes are manifested in multiple behavioral traits of both adults and larvae [33,34]. While the effect of this polymorphism on tunneling or embedding behavior has not been reported, it is not implausible that shorter tunnels evolved as correlated response to selection on reducing the locomotion while foraging.

It is interesting that another aspect of the construction behavior—embedding of the pupa—did not become less frequent in the course of adaptation to the larval malnutrition regime, even though embedding requires at least a short tunnel. This could mean that component of the tunneling behavior consumes more time or energy than embedding [30], or that the embedding behavior retained some adaptive advantage under the conditions imposed during the experimental evolution. The fact that the changes in tunnel length evolved independently of embedding frequency suggests that these two aspects of building behavior are controlled in part by different genetic loci. An analogous genetic independence of different elements of building behavior—entrance tunnel length and presence of an escape tunnel—has been reported in *Peromyscus* mice [35,36]. This modular nature of building behavior is thought to facilitate the complexity and evolutionary diversification of animal-made structures [2].

The adaptive significance of embedding and tunneling behaviors is largely unknown. Contrary to what one might expect, a pupal parasitoid has been shown to prefer ovipositing in embedded *Drosophila* pupae rather than those situated on the surface, possibly to benefit from protection embedding confers against other environmental factors [20]. Embedding may protect the pupa from desiccation and temperature fluctuations [19]. Tunneling may offer the same protection to pre-pupation larvae, while also sheltering them from some predators (the parasitoid result mentioned above notwithstanding) and cannibalistic conspecifics [27]. However, this kind of protection should be conferred even by a short tunnel in which the larva could remain motionless while waiting for pupation to begin. In natural environments it may be advantageous to pupate some distance away from the food patch—this could reduce exposure to molds, prevent the pupa from being drowned as the decomposing food liquefies [1,9,37], or reduce cannibalism [27]. Tunneling for a relatively long distance might help the larva to find a good place for pupation away from the food patch while remaining somewhat protected from the elements and the enemies. However, the fact that the behavior has been retained by strains maintained over many years in the laboratory suggests that it might confer some benefits even under laboratory culture conditions.

Irrespective of its the ecological significance, finding a facultative, variable and simple building behavior with quantifiable extended phenotype in a model system opens the way for research into the sensory, neural and genetic basis of this behavior.

## Methods

### Fly stock and maintenance

Canton S is an inbred strain maintained in the laboratory for several decades; Valais, an outbred strain derived from nature in Switzerland (2006). The selected and control populations were all derived from the same base population that was generated by mixing four populations of *D*. *melanogaster* that were originally founded by several hundred flies caught in Basel (Switzerland) in 1999 and maintained in the laboratory for about 100 generations [23,38]. They base population should thus be well adapted to the laboratory conditions prior to the start of the experiment. The selected populations were reared on poor larval food for 150 to 160 generations and in parallel the control populations were reared on standard food (15 g agar, 30 g sucrose, 60 g glucose, 12.5 g dry yeast, 50 g cornmeal, 0.5 g MgSO_4_, 0.5 g CaCl_2_, 30ml ethanol, 6 ml propionic acid and 1 g nipagin per liter of water) [23]. Both regimes were maintained at a density of 200 eggs/30 ml food, the adults were collected 14 days after egg laying except when not enough were collected, thus imposing selection on fast development, especially on poor food where development was generally slower than on standard food. The poor food contained ¼ of the amounts of sugars, yeast and cornmeal of the standard food described above. Prior to the assays reported here, all populations were reared on standard food for two generations to remove effects of maternal environment [39]. All experiments were carried out at 25°C, 60% humidity and 12:12 light cycle unless mentioned otherwise.

### Observation and quantification of tunneling and embedding

Observation and quantification of the pre-pupation tunneling behavior was done in large square (243 x 243 x 18 mm) Perspex plates (NUNC Bio-Assay Dishes) lined with a layer of agarose (2% agar w/v unless specified otherwise); 0.1% nipagin was added to prevent fungal growth. After the agarose solidified, a central circular area (radius 25mm) was removed and refilled with standard food unless mentioned otherwise. The plates were seeded with 100 eggs per strain. The behavior of wandering and tunneling larvae was video recorded and photographed until they pupated, with a Canon 7D DSLR camera mounted on a Leica stereo microscope.

To obtain quantitative data, for each plate the total number of pupae and the pupating position (embedded or nonembedded) of each pupa was scored. All the tunnels dug by the wandering larvae in the agar substrate of each plate were traced onto a single transparency sheet. The total tunnel length was then determined by digitizing these traces and measuring them using Image J software. The positions of all pupae on the plate were similarly traced onto a transparency sheet; the number of pupae in each concentric one centimeter circle around the food was counted to calculate the mean pupation distance. To study the relationship between embedding and developmental time, we set up eight assay plates. The number of larvae pupating and its position (exposed or embedded) was recorded daily.

### Building behavior in experimentally evolved populations

The larval building behaviors in the selected and control populations were assayed as described above. The assay was carried out in three blocks. For each block 12 plates were set-up (one for each of the six selected and six control populations) and incubated for eight days. In addition to comparing larvae of the selected and control lines raised on standard food, in a separate experiment we tested weather larval food affects the building behavior of the larvae from selected populations. In the latter assay the central zone of half of the assay plates contained poor rather than standard food and each of the selected population was assayed on one plate of each food type.

### Larval tunneling effort

The ‘tunneling effort’ put by the selected and control populations in digging was measured as the time taken for 30 cephalopharyngeal (mouth hook) retractions [40,41] in tunneling larvae (completely burrowed but still digging), in a separate assay. Two assay plates per population were set-up and six days later the plates were closely examined, eight tunneling larvae per plate were haphazardly chosen and their scleratic retraction-rate within the tunnel was determined under a dimly lit stereo-microscope (larvae abandon tunneling under bright light).

### Period between larval wandering forays and pupariation

Two assay plates were set up for each of the six selected and six control populations were setup. Wandering larvae from both regimes found on the agar arena at least 1.5 cm away from the central food zone were collected from the plate cultures every 4 hours, between 149–177 hours after oviposition. The collected larvae were transferred into fresh Petri plates lined with agar and the number of pupae that developed was scored every four hours. These data were used to estimate the time elapsed between the time when the larva was first collected outside of the food zone and their pupariation.

### Statistical analysis

The proportion data for eggs that successfully pupated and proportion of embedded pupae were arcsine square-root transformed prior to analysis. Most experiments were analyzed using mixed-model analysis of variance in JMP *v*. 8. The selection regime was included as a main effect while the replicated populations were nested within the selection regime and treated as a random effect. The population thus constituted the basic unit of replication. In assays done in several blocks, the block was also treated as a random effect.

For the experiment studying the period between collecting larvae outside of the food and their pupariation we used the larvae's age at the moment of collection as a covariate. The proportion of larvae that pupariated in this experiment were analysed with a logistic ANCOVA, with regime as the main effect, population nested within regime as a random effect, and age at collection as a covariate; this analysis was implemented using PROC GLIMMIX of SAS v. 9.3.

## Supporting Information

S1 Fig Effect of agar concentration on tunneling behavior. (PDF)

S1 Video This video shows a prepupal larva preparing the exit of its tunnel for embedding itself. The larva initially scrapes the agar around the tunnel exit while still retaining its posterior end within the tunnel it dug. The scraped agar eventually becomes mushy and solidifies as the larva pupates by embedding.
(MP4)
