# Supplementary material for: Prepupal Building Behavior in Drosophila melanogaster and Its Evolution under Resource and Time Constraints
Source: PLoS One. 2015 Feb 11;10(2):e0117280. doi: 10.1371/journal.pone.0117280 (PMC4324899; doi:10.1371/journal.pone.0117280)
Supplement: S1 Fig — (PDF) [file pone.0117280.s001.pdf]

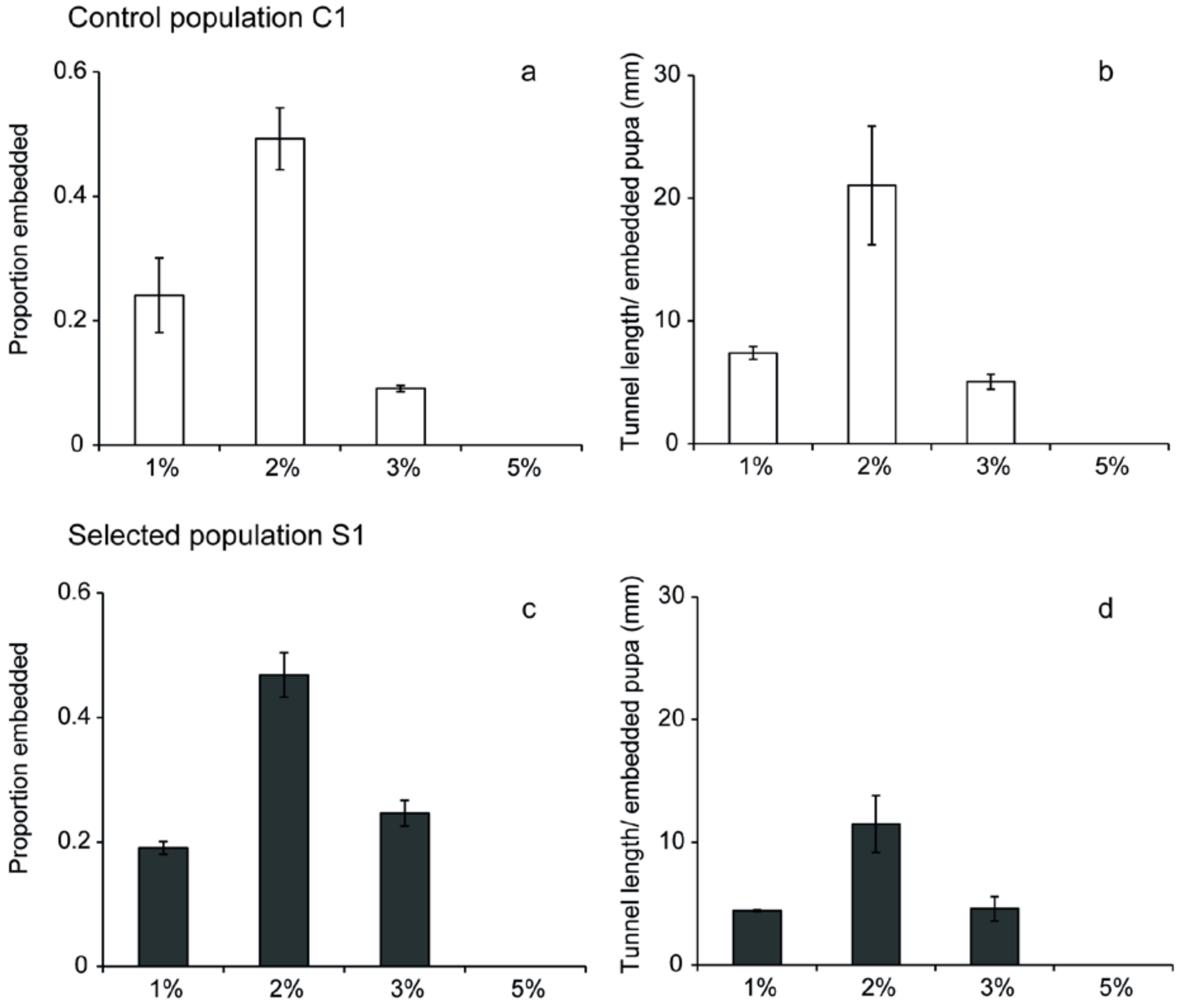

Supplementary Figure S1. Effect of agar concentration on tunneling.

Larval building behavior was assayed for a control population (C1) on plates with a range of agar concentrations (1 %, 2 %, 3 % and 5 %) in the wandering arena. (a) the proportion of pupae that embedded and (b) the average tunnel length of an embedded pupa (means ± CI) were both affected by agar concentration (embedding: $F_{2, 6} = 20.5$, $p = 0.0021$; tunnel length: $F_{2, 6} = 35.9$, $p = 0.0005$). A similar effect of agar concentration was observed when a selected population (S1) was assayed for (c) embedding and (d) tunneling behavior (embedding: $F_{2, 6} = 36.6$, $p = 0.0004$; tunnel length: $F_{2, 6} = 29.8$, $p = 0.0008$). Since neither embedding nor tunneling was recorded on 5% plates, it was excluded from the analysis; N = 3 assay plates per agar concentration per population.
